# The Unreliability of the Microscope in the Diagnosis of Malignant Disease

**Published:** 1902-09

**Authors:** C. Hamilton Whiteford

**Affiliations:** Medical Officer to the Provident Branch of the Plymouth Public Dispensary


### THE UNRELIABILITY OF THE MICROSCOPE IN THE DIAGNOSIS OF MALIGNANT DISEASE.

BY

C. Hamilton Whiteford, M.R.C.S., L.R.C.P.,

*Medical Officer to the Provident Branch of the Plymouth Public Dispensary.*

---

At the present time, when the whole subject of malignant disease is being reviewed in the light of recent knowledge and research, it may not be out of place to consider the value of the microscope, which has for many years been regarded as an essential and indispensable adjunct to diagnosis. In making the following criticisms, I wish it to be distinctly understood that it is the judgment, not the *bona fides*, of the microscopist which I mistrust.

Ten years ago I commenced surgical practice with a firm belief in the value of the microscope in the diagnosis of malignant disease, such belief being the natural result of my training as a student. From January, 1892, up to the present time I have seen the value of the microscope repeatedly and extensively tested in the diagnosis of all the more common varieties of malignant tumour. This process has resulted in a series of shocks to my previously firmly-rooted belief, and the only conclusion which I am able to draw from my experience is, that the microscope for purposes of differential diagnosis is totally unreliable. One of the earliest cases was that of a simple adenoma of the thyroid gland, which had been easily removed by enucleation. The growth was examined microscopically by a recognised authority, and reported to be undoubtedly malignant. Nothing in the naked-eye appearances of the growth or the after-history of the case supported this astounding piece of information. Similar equally erroneous diagnoses have repeatedly come under my observation.

Time after time I have seen the microscopical diagnosis of carcinoma, sarcoma, and inflammatory swelling absolutely disproved by the after-history and course of the disease. It is hardly to be wondered at that the microscope should thus fail in the diagnosis of a disease, the cause of which is at present unknown, notwithstanding the discovery of cancer parasites of late been reported almost weekly. The microscopist fails because he is trying to achieve what is, with our present knowledge, an impossibility. The surgeon is naturally reluctant to relinquish a belief in which he has been reared, but unless a particular article of surgical faith is justified by results its further logical tenure becomes impossible. This, I contend, is the case with the belief in the microscope for the diagnosis of cancer. In cases in which the clinical appearances and history, aided by the naked-eye appearances on section, fail to settle the diagnosis, I have generally found the microscopist's report to be of no assistance.

Mistrust of the microscope as a court of appeal, in which the nature of a tumour can be decided once and for all, is on the increase, of which the following expressions of opinion are evidence :—

The *Lancet* has summarised Mr. Jonathan Hutchinson's [1] views in an annotation entitled, "The Microscope and the Early Stages of Cancer," as follows :—

"That microscopic examination is a valuable aid in the diagnosis of cancer and in doubtful cases a decisive test is generally held. Such is not the view of Mr. Hutchinson. In a recent number of the *Archives of Surgery* he insists that the microscope can give no aid in the recognition of the precancerous stages of cancer. For a long time he has been a warm advocate of early operations and in certain cases of their performance before absolute certainty in diagnosis can be felt. He states that in well-pronounced conditions microscopic examination is not needed and in doubtful ones it cannot assist. Characteristic appearances in the mode of growth and the manner of ulceration are almost always obvious to the instructed eye before the histological conditions have become such that they can be trusted. Thus, according to Mr. Hutchinson, in their early stages cancerous growths are not histologically cancer; chronic inflammatory changes almost invariably precede for a

[1] *Lancet*, 1898, i. 1132.

longer or shorter period those which are definitely malignant. A negative result with the microscope often begets a false security and leads to a loss of time which it is impossible to retrieve. Mr. Hutchinson finds that this is constantly occurring; in the previous month he saw four such cases. A man had a large and most characteristic epithelial cancer in his soft palate in a stage almost too late for excision. Eighteen months previously a portion had been cut away which on microscopical examination was pronounced not to be cancer. Mr. Hutchinson asks, 'Would it not have been better to have removed the whole?' In a case of ulcer of the tongue the patient was assured as the result of microscopical examination that he was not suffering from cancer, but two months later the contrary was proved. On one occasion Mr. Hutchinson removed the whole tongue for what he believed to be a broken-down cancerous mass which had suppurated. An experienced pathologist who examined the specimen assured him that there was no cancer but simply an abscess cavity. Within a few months, however, the glands enlarged, and in less than a year the patient died. In a case of cancer in the palm of the hand occurring after prolonged administration of arsenic, microscopic sections were made which were examined by various authorities in America, in this country, and on the continent, with the result that most diverse opinions were given. Several insisted that the disease was syphilis and not cancer."

Sir William M. Banks,[1] in the *Lettsomian Lectures for the Year 1900*, in discussing the differential diagnosis between chronic interstitial mastitis and cancer, after a lifetime's experience in the surgery of the breast, says:—"How is this to be known? By cutting through any suspicious mass or nodule. I am quite sure that the section which an experienced surgeon makes with a knife through a fresh piece of carcinoma will enable him to give a far more decided and certain opinion than anything which the microscopist can give. Not that for a minute I would speak disparagingly of the value of microscopical examination. When it coincides with the naked-eye view of the clinician so much the better, but when they differ I hold

[1] *Lancet*, 1900, i. 846.

that the history of the tumour and the look and feel of the fresh section constitute the most trustworthy guide."

Mr. J. Knowsley Thornton,[1] in a Presidential Address to the Harveian Society in the year 1897, said: "Though formerly an enthusiast with the microscope, he had come to regard the knowledge which it gave as over-rated, especially in regard to new growths. If he had to choose between the microscope and clinical observation in deciding what was malignant and what benign, he would rather be guided by the latter, though the microscope was useful in suggestion and in confirmation. It should not be the arbiter in doubtful cases."

Dr. H. D. Rolleston and Mr. H. W. J. Marks,[2] in an article on "Primary Malignant Disease of the Supra-renal Bodies" (published in the *American Journal of the Medical Sciences* for October, 1898), point out that it is almost impossible to differentiate histologically an adenoma disorganised by hemorrhage from a primary malignant growth. Hence they prefer to rely for diagnosis of malignancy on metastasis and invasion of surrounding parts, and not on the microscope.

Mr. Marmaduke Sheild,[3] in a lecture on "Mammary Cancer," says: "It may seem heretical to state that the naked-eye appearances as exposed on exploratory incision are, in my own opinion, more likely to lead the surgeon right than are the results of microscopical examination. I have always found that when I have been in doubt between inflammatory tissue and early cancer the same doubt occurs to the microscopist if only he possesses the somewhat rare attributes of a sceptical mind associated with accuracy of observation. On the other hand, I have known serious prognostical difficulties to arise from relying too certainly upon dubious microscopical appearances. I cannot recommend these observations too strongly to your serious consideration." Later in the same lecture Mr. Sheild says:—"At the same time I am coming more and more to the conclusion, which I fear many of my audience will term retrograde, to depend more and more upon naked-eye appearances and less upon microscopical, and unless a

[1] *Brit. M. J.*, 1897, i. 269.

[2] *Lancet*, 1898, ii. 1283. [3] *Ibid.*, 1902, i. 643.

nodule of cancer be found showing the typical appearances on section to the naked-eye to be hyper-cautious regarding any positive diagnosis made by the microscope only."

At a meeting of the Royal Academy of Medicine in Ireland on January 22, 1897, Mr. Myles,[1] in a discussion on "Cancer of the Tongue," said: "Microscopic examinations of scrapings or of fresh sections of the tongue were absolutely unreliable. Professor Hamilton of Aberdeen brought under the notice of the profession the changes that occurred in the tongues of old people, and produced microscopic sections of the tongues of persons over fifty-five years of age, and every one of these presented the identical appearances that were usually associated with epithelioma. He placed side by side with them sections of clinical epithelioma and the most expert pathologists were unable to distinguish between the two."

My present position is this—the microscope having failed me, I am thrown back on the relatively more reliable naked-eye appearances.

If the clinical history and naked-eye appearances on fresh section indicate that the growth is malignant, but under the microscope the growth appears innocent, I regard the growth as malignant.

If the naked-eye appearances are innocent, but the microscopic appearances malignant, I regard the growth as being innocent.

In other words, when the naked-eye and microscopic appearances differ I base my diagnosis and treatment on the clinical history and naked-eye appearances on fresh section, because I have found them to be the more reliable.

[1] *Lancet*, 1897, i. 456.

